# Supplementary material for: Resolving the full spectrum of human genome variation using Linked-Reads
Source: Genome Res. 2019 Apr;29(4):635–45. doi: 10.1101/gr.234443.118 (PMC6442396; doi:10.1101/gr.234443.118)
Supplement: Supplemental Material [file supp_gr.234443.118_Supplemental_longranger-2.2.zip › longranger-2.2.tar.bz2!/lr2.2-public/lib/stan/cmdstan/stan/doc/api/html/sub__col_8hpp_source.html]

Stan: src/stan/math/matrix/sub\_col.hpp Source File


|  |  |
| --- | --- |
|  | Stan  2.5.0  probability, sampling & optimization |


- Main Page
- Related Pages
- Namespaces
- Classes
- Files

- File List
- File Members

All Classes Namespaces Files Functions Variables Typedefs Enumerations Enumerator Friends Macros Pages

- src
- stan
- math
- matrix

sub\_col.hpp

Go to the documentation of this file.

1 #ifndef STAN\_\_MATH\_\_MATRIX\_\_SUB\_COL\_HPP

2 #define STAN\_\_MATH\_\_MATRIX\_\_SUB\_COL\_HPP

3

4 #include <stan/math/matrix/Eigen.hpp>

5 #include <stan/math/error\_handling/matrix/check\_row\_index.hpp>

6 #include <stan/math/error\_handling/matrix/check\_column\_index.hpp>

7

8 namespace stan {

9  namespace math {

10

19  template <typename T>

20  inline

21  Eigen::Matrix<T,Eigen::Dynamic,1>

22  sub\_col(const Eigen::Matrix<T,Eigen::Dynamic,Eigen::Dynamic>& m,

23  size\_t i, size\_t j, size\_t nrows) {

24  stan::math::check\_row\_index("sub\_col(%1%)",i,m,"i",(double\*)0);

25  if (nrows > 0)

26  stan::math::check\_row\_index("sub\_col(%1%)",i+nrows-1,m,"i+nrows-1",(double\*)0);

27  stan::math::check\_column\_index("sub\_col(%1%)",j,m,"j",(double\*)0);

28  return m.block(i - 1,j - 1,nrows,1);

29  }

30

31

32  }

33 }

34

35 #endif

check\_column\_index.hpp

stan::math::check\_column\_index

bool check\_column\_index(const char \*function, size\_t i, const Eigen::Matrix< T\_y, R, C > &y, const char \*name, T\_result \*result)

Return true if the specified index is a valid column of the matrix.

**Definition:** check\_column\_index.hpp:25

check\_row\_index.hpp

stan::math::check\_row\_index

bool check\_row\_index(const char \*function, size\_t i, const Eigen::Matrix< T\_y, R, C > &y, const char \*name, T\_result \*result)

Return true if the specified index is a valid row of the matrix.

**Definition:** check\_row\_index.hpp:25

Eigen.hpp

stan::math::sub\_col

Eigen::Matrix< T, Eigen::Dynamic, 1 > sub\_col(const Eigen::Matrix< T, Eigen::Dynamic, Eigen::Dynamic > &m, size\_t i, size\_t j, size\_t nrows)

Return a nrows x 1 subcolumn starting at (i-1,j-1).

**Definition:** sub\_col.hpp:22


---

[ Stan Home Page ]

*© 2011–2014,
Stan Development Team.*
